# Anticandidal Properties of *Launaea sarmentosa* among the Salt Marsh Plants Collected from Palk Bay and the Gulf of Mannar Coast, Southeastern India

**DOI:** 10.3390/antibiotics13080748

**Published:** 2024-08-09

**Authors:** Smriti Das, Karuppannagounder Rajan Priyanka, Kolandhasamy Prabhu, Ramachandran Vinayagam, Rajendran Rajaram, Sang Gu Kang

**Affiliations:** 1 Department of Marine Science, School of Marine Sciences, Bharathidasan University, Tiruchirappalli 620 024, Tamil Nadu, India; dassmriti874@gmail.com (S.D.); priyanka.kr@bdu.ac.in (K.R.P.); prabhu.k@bdu.ac.in (K.P.); 2 Department of Biotechnology, Institute of Biotechnology, College of Life and Applied Sciences, Yeungnam University, 280 Daehak-Ro, Gyeongsan 38541, Gyeongsangbuk-do, Republic of Korea

**Keywords:** phytochemical, GC-MS, seasonal variation, *Launaea sarmentosa*, *Candida* spp.

## Abstract

Tidal wetlands, commonly known as salt marshes, are highly productive ecosystems in temperate regions worldwide. These environments constitute a unique flora composed primarily of salt-tolerant herbs, grasses, and shrubs. This study investigated the therapeutic properties of ten salt marsh plants collected mainly from Palk Bay and Mannar Gulf against Candida disease. This study examined the changes in natural plant products associated with their anti-Candida growth activity during two distinct seasonal changes—monsoon and summer. The potential of the salt marshes to inhibit the growth of five different *Candida* strains was assessed using four solvents. In phytochemical analysis, the extracts obtained from a *Launaea sarmentosa* exhibited the highest results compared to the other plant extracts. Fourier transform infrared spectroscopy revealed 12 peaks with alkane, aldehyde, amine, aromatic ester, phenol, secondary alcohol, and 1,2,3,4-tetrasubstituted. Gas-chromatography–mass spectrometry detected 30 compounds. Cyclotetracosane, lupeol, β-amyrin, and 12-oleanen-3-yl acetate showed the highest peak range. In particular, plant samples collected during the monsoon season were more effective in preventing Canda growth than the summer plant samples. In the monsoon season, the salt marsh plant extracted with ethyl acetate showed a high anti-Candida growth activity, while in the summer, the acetone extract exhibited a higher anti-Candida growth activity than the other solvents. The hexane extract of *L. sarmentosa* showed the highest inhibition zone against all Candidal strains. Furthermore, compounds, such as β-amyrin, lupeol, and oxirane, from the hexane extract of *L. sarmentosa* play a vital role in anti-Candida activity. This paper reports the potential of tidal marsh plant extracts for developing new antifungal agents for Candida infections.

## 1. Introduction

*Candida*, a yeast under the kingdom fungi, is a typical organism found all over the body, including the oral cavity and mucous membrane of reproductive and gastrointestinal organs. Up to 80% of people with good health have been susceptible to the most prevalent fungal infections, such as candidiasis. The severity of invasive candidiasis can range from mild candidemia with limited symptoms to a life-threatening condition of fulminant sepsis, where the associated mortality rate exceeds 70% [1]. Among the 150 distinct varieties of *Candida* spp., few have the potential to infect humans. Among these, *Candida albicans* is the most pathogenic [2] and one of the crucial fungal pathogens in humans, which is normally not harmful but a symbiotic organism. For individuals with compromised immune systems, it is an opportunistic pathogen that is responsible for painful mucosal infections, such as vaginitis for women and oropharyngeal candidiasis for AIDS patients. Several steps are essential for preventing the spread of *Candida*, including maintaining good hygiene and utilizing appropriate infection control measures in healthcare settings [3,4].

The marine environment has an astonishing array of life forms, ranging from microscopic to massive organisms. Marine ecosystems harbor over 80% of the world’s species, with many still undiscovered [5]. Marine wetlands are a prosperous and significant ecosystem that delivers a variety of cultural and environmental advantages and are now categorized as an ecosystem servicer, which provides services, namely water quality maintenance, C-sequestration, atmospheric gas regulation, shoreline protection, and maintaining distinctive indigenous biota [6]. Nevertheless, these ecosystems comprise only 1.5% of the planet’s surface, while providing 40% of the ecosystem services worldwide [7]. Recently, wetlands have attracted increasing attention from ecology and conservation standpoints. Halophytes, a wetland vegetation that can thrive and grow in extreme salt environments, are globally distributed and serve as a great ecosystem with huge productivity, mainly in equatorial regions. As a transitional region, salt marshes can be found among terrestrial and marine ecosystems [8]. Salt marsh plants have long been used for medicinal purposes owing to their unique biochemical properties and adaptations to harsh saline environments. Salt marsh plants have been used in various industries, such as the food and cosmetics industries, owing to their bioactive compounds and tolerance adaptability [8]. Many salt marsh plants have promising antioxidant, antimicrobial, anti-inflammatory, and anticancer properties, with additional recreational and pharmaceutical uses. Recently, researchers have been exploring the potential of salt marsh plants as sources of novel compounds with biological activities. Several studies have evaluated anticandidal compounds in terrestrial plants [9,10], but no compound-level studies have been conducted in salt marsh plants. Hence, this study focused primarily on the anticandidal properties of different salt marsh plants collected from southeastern coastal India.

## 2. Results and Discussion

The results highlight the phytochemical constituents and anticandidal potential of various salt marsh plant extracts collected during two distinct seasons (monsoon and summer) and tested against five distinct Candidal strains using four solvents—ethyl acetate, acetone, hexane, and methanol. During sample collection, regular monitoring of salt marsh plants was carried out to determine the availability of the samples in the sampling sites. The resulting activity based on the seasonal variation is reported in table form. Ethyl acetate exhibited promising activity for all plant extracts against most of the *Candida* strains, but the hexane extract of *L. sarmentosa* showed higher activity against all *Candida* species. 

### 2.1. Phytochemical Screening

This study examined the most active solvent extracts from salt marsh plants among 10 samples. The extracts showed the maximum efficacy when the acetone, ethyl acetate, and hexane extracts derived from *H. curassavicum*, *S. portulacastrum*, *S. maritima*, and *Launaea sarmentosa* were used. The acetone and ethyl acetate extract of *H. curassavicum*, *S. portulacastrum*, *S. maritima*, and *L. sarmentosa*, as well as the hexane extract of *Launaea sarmentosa*, exhibited various phytochemical components with significant importance in the pharmaceutical field. The phytochemical components tested were alkaloids, glycosides, flavonoids, steroids, phenolic groups, saponins, coumarin, quinines, tannins, and terpenoids, as shown in Table 1 and Figures S5–S7. The extracts obtained from *L. sarmentosa* showed maximum activity for all the chemical constituents compared to the other plant extracts. The ethyl acetate extract of *L. sarmentosa* contained almost all of its chemical constituents. Kader et al. [11] conducted a phytochemical screening and antimicrobial evaluation of various fractions of the ethanolic extract of *L. sarmentosa* to search for possible candidates for killing multidrug-resistant microbes. They reported the presence of alkaloids, flavonoids, glycosides, steroids, terpenoids, phenolic compounds, and carbohydrates in the ethyl acetate extract of *Launaea sarmentosa*, but reported the absence of tannins and saponins. In contrast, in this investigation, the ethyl acetate extract of the same species did contained tannin. After *L. sarmentosa*, *S. maritima* exhibited the maximum presence of all the constituents. Among all the extracts of *S. maritima*, the acetone extract showed the maximum result for almost all constituents. Akbar et al. [12] explained that the ethyl acetate extract of *H. curassavicum* confirmed the presence of alkaloids, saponins, tannins, flavonoids, steroids, terpenoids, glycosides, phenols, and coumarin but the absence of quinone. In contrast, the current study confirmed the presence of quinone, but saponin was absent in the *H. curassavicum* extract. Among all the phytochemical constituent alkaloids, tannin and terpenoids are present in every solvent extract. On the other hand, saponin exhibited the lowest level among all extracts. During this investigation, phenolic groups were observed in every crude extract, indicating the antifungal activities in these plants. 

### 2.2. Anticandidal Assay

**Figure 1:**
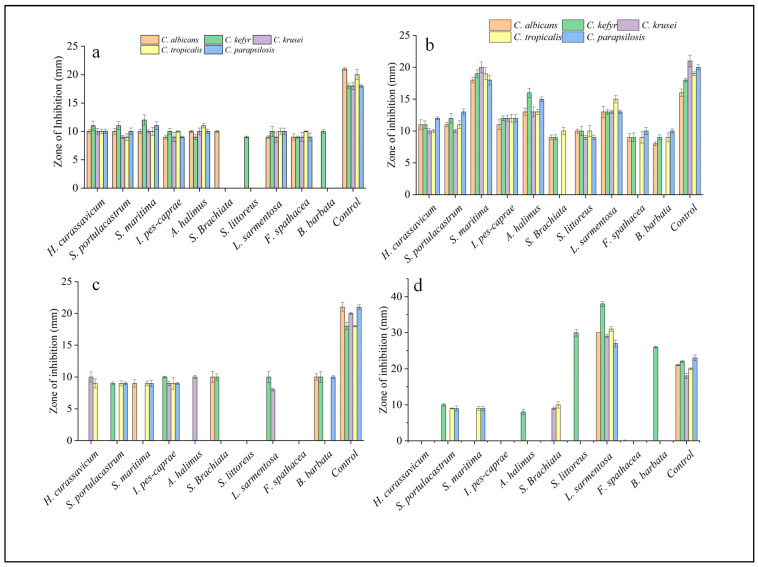
Anticandial properties of salt marsh plants exhibited as the zone of inhibition against Candidal strains during the monsoon season ((**a**)—Acetone; (**b**)—Ethyl Acetate; (**c**)—Methonal and (**d**)—Hexane).

**Figure 2:**
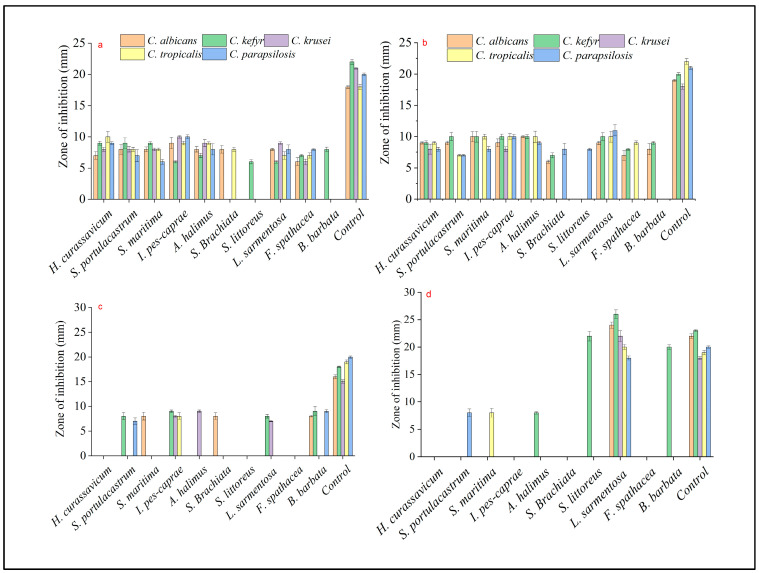
Anticandidal properties of salt marsh plants exhibiting zone of inhibition against Candidal strains during the summer season ((**a**)—Acetone; (**b**)—Ethyl acetate; (**c**)—Methanol; and (**d**)—Hexane).

Regarding the samples obtained during the monsoon period, ethyl acetate and acetone extracts exhibited the highest activity, followed by methanol and hexane (Figures S1–S4). The extracts obtained from ethyl acetate for all the samples showed promising results. Among them, *S. maritima* demonstrated the maximum inhibition zone against *C. krusei* (20 ± 0.86 mm), followed by *Atriplex halimus* against *C. kefyr* (16 ± 0.7 mm*)* and *L. sarmentosa* against *C. tropicalis* (15 ± 0.61 mm). Mohamed et al. [13] expressed *S. maritima* as a traditional medicine used to treat malaria. *Atriplex halimus*, as reported by El-Aasr et al. [14], exhibited a wide-ranging antimicrobial effect through the isolation of four flavanol glycosides, isorhamnetin 3-O-β-D-rutinoside (narcissin), syringetin 3-Oβ-D-rutinoside, artiplexoside A, and syringetin 3-O-β-D-glucopyranoside, in which artiplexoside A is an anticandidal agent. Kader et al. [11] reported that the ethyl acetate extract of *L. sarmentosa* has a potential antifungal activity (13 ± 0.87 mm against *C. albicans*). The acetone extract of *S. maritima* showed the highest activity against *C. kefyr* (12 ± 0.9 mm); the acetone extract of *S. littoreus* showed minimum activity against all pathogenic strains followed by *B. barbata.* Although hexane showed less activity, the extract from *Launaea sarmentosa* showed an impressive result with a maximum inhibition zone against *C. kefyr* (38 ± 0.71 mm) and with minimum inhibition against *C. parapsilosis* (27 ± 0.94 mm) (Table 2). Fluconazole was used as the positive control. Based on the present investigation, compared to the positive control, the hexane extract of *L. sarmentosa* showed a higher zone of inhibition. The maximum zone of inhibition in fluconazole was 23 ± 0.8 mm, whereas *L. sarmentosa* showed 29 ± 0.46–38 ± 0.71 mm inhibition against the tested *Candida* strains. Kader et al. [11] reported that *L. sarmentosa* exhibited antibacterial and antifungal potentiality with the presence of several phytochemical constituents, such as flavonoids, alkaloids, steroids, tannins, and phenolic compounds, which demonstrates the possibility for the formulation of innovative and potent antimicrobial treatments. In addition, the hexane extract of *S. littoreus* and *B. barbata* exhibited a promising result against *C. kefyr*. The activity sequence of the extract taken in the monsoon was ethyl acetate > acetone > methanol > hexane (Figure 1). The summer sample exhibited less activity than the monsoon sample. In summer, the acetone solvent extract exhibited maximum potential, followed by ethyl acetate, methanol, and hexane (Table 3). The activity sequence of solvent extracts in the summer season was acetone > ethyl acetate > methanol > hexane (Figure 2).

**Figure 3:**
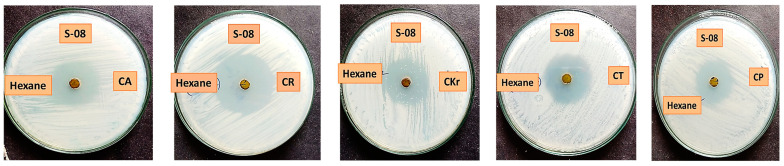
Anticandidal activity of saltmarsh plant *Launaea sarmentosa* extract exhibits the highest inhibition (CA—*Candida albicans*; CR—*Candida kefyr*; CKr—*Candida krusei;* CT—*Candida tropicalis*; CP—*Candida parapsilosis*).

Among all the acetone extracts, *H. curassavicum* showed the maximum inhibition zone against *C. tropicalis* (10 ± 0.87 mm), while *S. littoreus* barely showed any activity. Ghori et al. [15] examined *H. curassavicum*, an important medicinal plant used traditionally for various purposes, such as for skin diseases, poisonous bites, gout, inflammation, and menstrual disorders. In contrast, this plant has significant biological properties, such as antiviral, wound healing, antitumor, phytotoxicity, and anti-inflammatory activities, with important pharmacogenetic properties. Ethyl acetate showed good results for almost all the Candidal pathogens. Among them, the extract of *L. sarmentosa* showed the maximum inhibition zone against *C. parapsilosis* (11 ± 0.9 mm), while *S. Brachiata* exhibited the lowest inhibition zone agains*t C. albicans*. The *Spinifex littoreus* extract barely showed any activity. The methanol solvent extract showed an average activity against all the Candidal pathogens. Although hexane showed less activity overall, the *L. sarmentosa* extract combined with hexane showed the highest activity among the four solvent extracts (summer sample) with a maximum zone of inhibition (26 ± 0.78 mm) against *C. kefyr. In contrast*, the same extract showed the minimum inhibition zone against *C. parapsilosis* (Figure 3). Prapasanobol [16] reported that *L. sarmentosa has* potential anti-mycobacterium tuberculosis activity with alkaloids, steroids, and flavonoids. The hexane extract of *L. sarmentosa* was examined based on the anticandidal analysis. The activity sequence of the extract in the monsoon season was ethyl acetate > acetone > methanol > hexane, and the activity sequence of the solvent extract in the summer season was acetone > ethyl acetate > methanol > hexane. The monsoon samples showed more activity than the summer samples.

### 2.3. FT-IR Spectroscopy

**Figure 4:**
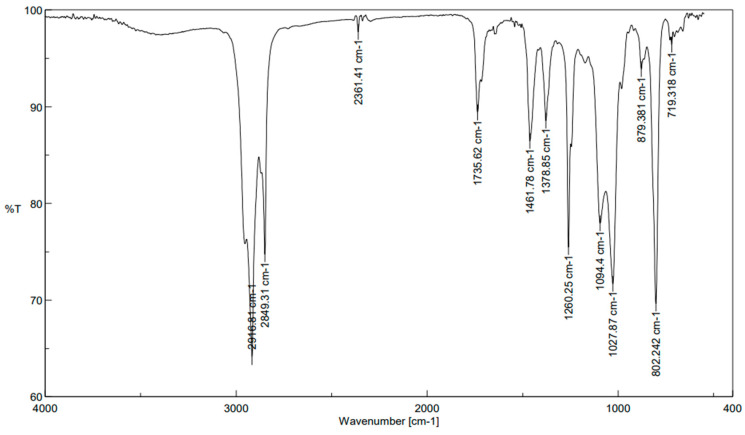
FT−IR spectrum showing the peaks obtained from the hexane extract of salt marsh *Launaea sarmentosa*.

The spectrum of the hexane extract of *L. sarmentosa* exhibited 12 peaks. The first peak at 2916.81 cm^−1^ was assigned to the N–H stretching vibration, which shows amine salt. The C–H stretching and C–H bending vibrations were observed at 2849.31 and 1461.78 cm^−1^, respectively; the presence of flavonoids and tannins was signaled by these bands [17]. The band region of other peaks at 802.242, 1027.87, and 1260.25 cm^−1^ were attributed to the 1,4-disubstituted or 1,2,3,4-tetrasubstituted, amine, and aromatic ester groups. Figure 4 and Table 4 show the peaks with the corresponding groups. The strong and sharp band, along with the N–H stretching vibration, indicated the presence of aromatic and aliphatic amine groups.

### 2.4. GC-MS Analysis

**Figure 5:**
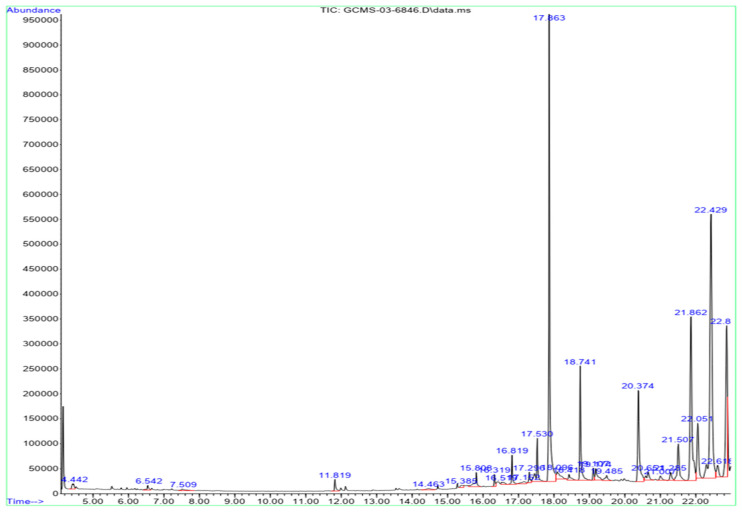
GC-MS showing the peaks obtained from the hexane extract of saltmarsh *Launaea sarmentosa*.

GC-MS was used to examine the composition of chemical constituents present in the hexane extract of *L. sarmentosa*. Figure 5 and Table 5 show the 30 active compounds identified from the hexane extract of *L. sarmentosa*. The major compounds characterized included β-amyrin, lupeol, oxirane, pentadecane, cyclotetracosane, and hexadecane, followed by cyclopropane, 2-bromo-1,1,3-trimethyl-2,6,10-trimethyl-, tetradecyl fluoride, 1-methoxy-3-(2-hydroxyethyl)nonane, cyclotrisiloxane, trimethyl[4-(1,1,3,3,-tetramethylbutyl) phenoxy]silane, heptadecane, hexadecane, and silane. Despite the insufficient data for *L. sarmentosa* species, Hanh et al. [18] examined the methanolic root extract of *L. sarmentosa*. They reported compounds such as α- and β-amyrin and Lupeol, similar to the current investigation. According to Melo et al. [19], the β-amyrin obtained from the wood resin of *Protium heptaphyllum* exhibits an inhibitory activity against acute pancreatitis. Ahsan et al. [20] obtained eicosane from Streptomyces, which helps to control tobacco leaf spot disease. Beema Shafreen et al. [21] used the molecular docking method to determine that eicosane emerged as a potent inhibitor for the growth of *Candida albicans*. The purified compounds octacosane and eicosane from *Marantodes pumilum* fraction A exhibited wound-healing properties in diabetic-induced animals [22]. 

## 3. Materials and Methods

### 3.1. Collection and Identification of Samples

**Figure 6:**
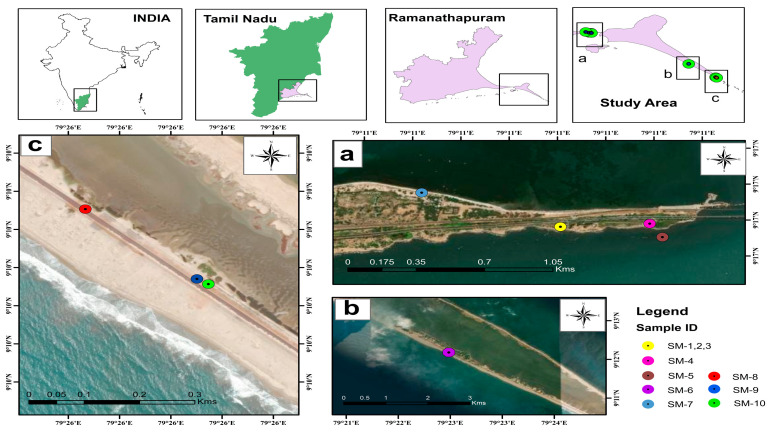
Sampling sites of salt marsh plants collected from Palk Bay and the Gulf of Mannar.

**Figure 7:**
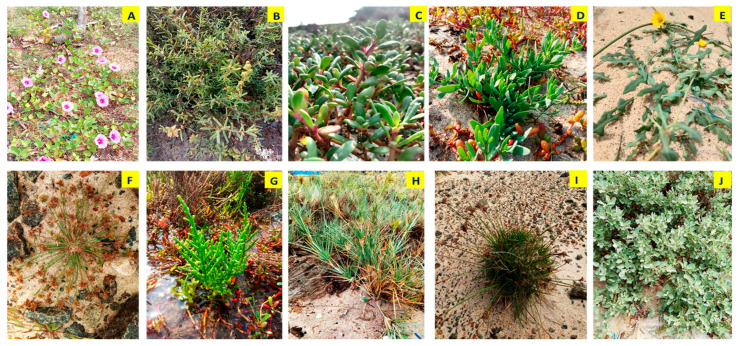
Salt marsh plants collected from Palk Bay and the Gulf of Mannar ((**A**) *Ipomoea pes-caprae*, (**B**) *Suaeda maritima*, (**C**) *Sesuvium portulacastrum*, (**D**) *Heliotropium curassavicum*, (**E**) *Launaea sarmentosa*, (**F**) *Bulbostylis barbata*, (**G**) *Salicornia brachiata*, (**H**) *Spinifex littoreus*, (**I**) Fim*bristylis spathacea*, and (**J**) *Artiplex halimus*).

Salt marsh plants were collected from the Gulf of Mannar and Palk Bay coastal region (Mandapam and Dhanushkodi), Southeast coast of India (Figure 6 and Table 6). The plant species were monitored periodically and samples were collected in two seasons—the monsoon (mid-June to December) and summer (March to May). Ten species of salt marsh plants were collected in eight sampling sites (Table 7). The collected samples comprised one coastal creeping herb and two estuarine grass species (tidal fringe wetland) identified through established guidelines outlined in the following manuals [18,23,24,25,26,27,28]. The collected samples included *Heliotropium curassavicum* L., *Sesuvium portulacastrum* (L.) L., *Suaeda maritima* (L.) Dum., *Ipomoea pes-caprae* (L.) R. Br., *Atriplex halimus* L., *Salicornia Brachiata* Roxb., *Spinifex littoreus* (Burm.f.) Merr., *Launaea sarmentosa* (Willd.) Kuntze, *Fimbristylis spathacea* Roth, and *Bulbostylis barbata* (Rottb.) C.B.Clarke (Figure 7).

### 3.2. Processing and Preparation of Samples

The collected salt marsh plants were washed thoroughly with seawater immediately after collection. The collected samples were brought to the laboratory and were continuously washed with fresh water to remove salt and other debris attached to them. They were allowed to dry in air at room temperature. The dried samples were weighed and ground with a grinder and stored in a sealed container for future purposes. To extract the finely ground salt marsh plant powder, four distinct solvents—methanol, acetone, ethyl acetate, and hexane were added to each flask. The solution was soaked in the solvents and agitated in an orbital shaker for 46 h. The extracts were filtered through Whatman filter paper and kept for evaporation [29].

### 3.3. Chemical, Strains, and Media

Analytical grade solvents, such as methanol, acetone, ethyl acetate, and hexane, were purchased from HI media. Whatman filter paper (Grade 1) was purchased from HI Media. Five *Candida* strains that were used through all experiments, including *Candida albicans*, *Candida parapsilosis*, *Candida kefyr*, *Candida krusei*, and *Candida tropicalis*, were obtained from the National Culture Collection of Pathogenic Fungi (NCCPF) for this anticandidal study.

### 3.4. Phytochemical Analysis

Initially, phytochemical screening was performed using the standard method [30,31,32,33,34,35,36].

#### 3.4.1. Alkaloid Detection (Wagner’s Test)

A 1 mL sample of plant extract was combined with one to two drops of Wagner’s reagent. The formation of a reddish-brown precipitate or coloration indicates the presence of alkaloid.

#### 3.4.2. Flavonoid Identification (Alkaline Reagent Test)

A 2 mL sample of plant extract was mixed with 2 mL of 5% NaOH, and 1 mL of 50% H₂SO_4_ was then added. The initial yellow coloration that fades or becomes colorless upon acidification indicates the presence of flavonoids.

#### 3.4.3. Cardiac Glycoside Assay (Keller–Killiani Test)

A 2 mL sample of the extract was combined with 0.5 mL glacial acetic acid containing FeCl_3_, and was layered with 1 mL of concentrated H₂SO_4_. The formation of deep blue coloration at the interface of the two liquids indicates the presence of cardiac glycosides.

#### 3.4.4. Phenolic Compound Detection (Ferric Chloride Test)

A 1 mL sample of the extract was mixed with 2 mL 5% FeCl_3_ solution. The development of dark blue coloration indicates the presence of phenolic compounds.

#### 3.4.5. Saponin Analysis (Froth Test)

A 5 mL sample of crude extract was combined with 20 mL of distilled water and was shaken vigorously for 15 min. The persistence of a foam layer on the liquid surface after agitation indicated the presence of saponins.

#### 3.4.6. Steroid (Salkowski’s Test)

A 1.5 mL sample of the extract was mixed with 1 mL chloroform and was layered with 1.5 mL of concentrated H₂SO_4_. Reddish-brown coloration at the interface between aqueous and organic phases indicates the presence of steroids.

#### 3.4.7. Tannin Detection (Ferric Chloride Test)

A 1 mL sample of the extract was combined with 1 mL of 10% FeCl_3_ solution. The formation of blue-black or brownish-green coloration indicates the presence of tannins.

#### 3.4.8. Terpenoid Assay (Salkowski Test)

A 5 mL sample of the extract was mixed with 2 mL of chloroform and was layered with 3 mL of concentrated H₂SO_4_. A reddish-brown coloration at the interface between aqueous and organic phases indicates the presence of terpenoid compounds.

#### 3.4.9. Coumarin Detection

A 1 mL sample of the extract was mixed with 1 mL of a 10% NaOH solution. The development of yellow coloration upon mixing indicates the presence of coumarin.

#### 3.4.10. Quinone 

A 1 mL sample of the extract was combined with 1 mL of concentrated H₂SO_4_. The formation of red coloration in the mixture indicates the presence of quinone.

### 3.5. Anticandidal Screening

All fungal strains were kept in Yeast Peptone Dextrose agar medium, which was prepared at a 1:2:1 ratio in glycerol stock for further studies. The effectiveness of suppressing *candida* cells with the help of salt marsh plant extract was initially assessed using the agar diffusion method [29]. Under an ascetic atmosphere, the sterilized plates of potato dextrose agar were prepared. The prepared plate was swabbed with the 24 h-cultured *Candida* strains. The crude extract obtained from the extraction of salt marsh was inoculated in the punctured wells (6 mm) and placed in the incubator at 37 °C for one day [4,29]. The anticandidal activity of the salt marsh extract was determined by measuring the zone of inhibition using the zone of inhibition scale. Triplicate measures were carried out to verify the effectiveness of salt marsh plant extracts. The MS Excel (Windows 11) application was used to convert the data of the triplicates into the mean and standard deviation [29]. Further investigations were conducted only on the particular samples based on the zone of inhibition obtained from the collected samples.

### 3.6. FTIR 

Salt marsh plants that exhibit maximum inhibition were processed and recorded using Fourier transform infrared (FTIR, JASCO FT/IR-6600) spectroscopy to analyze the functional groups in the selected salt marsh extracts. The extracts from salt marsh plants were dissolved in the required solvent, and the absorbance was recorded from 4000 to 400 cm^−1^ [37]. 

### 3.7. GC-MS

The compounds in the selected salt marsh plant sample were quantified using gas chromatography–mass spectrometry (GC-MS, Agilent, Santa Clara, CA, USA, GC 7890A/MS5975C). The peaks were detected with a column length of 30 m, an internal diameter of 0.25 mm, and a film thickness of 0.25 μm. The NIST library was used to identify the compounds present in the analyzed saltmarsh samples. The extracts from the salt marsh plants were dissolved with the required solvent, filtered using a GC filter, and were placed in a GC vial for further analysis. The chromatograph was run for 20 min, and the compounds eluted are reported in table and spectra form [38].

## 4. Conclusions

The different extracts from the collected salt marsh plants exhibit varying degrees of antifungal activity against Candida strains, with some showing consistent efficacy throughout different seasons. Phytochemical analysis showed that the extract of L. sarmentosa showed the highest activity compared to the other extracts. All plant extracts confirmed the strong presence of phenolic groups, indicating the antifungal activity of these crude extracts derived from plants. In the monsoon season, the ethyl acetate sample showed potential against five pathogenic strains. In summer, the acetone sample showed more activity than the other three solvents. Compared to the other solvent extracts tested, the hexane extract showed less activity, but in *L. sarmentosa*, the hexane extract exhibited the highest activity against all candidal strains, in both seasons. The evaporation of the volatile compound in excess heat is the main reason that the summer samples have a lower activity. Compounds such as β-amyrin, lupeol, and oxirane play a significant role in biological applications. These findings highlight the potential of these salt marsh plant extracts as sources for the further development of novel antifungal agents to combat azole-resistant *Candida* infections. Nevertheless, further studies will be needed to explore their full potential and elucidate the underlying mechanisms of action via in silico and in vivo analysis.

## Figures and Tables

**Table 1:** Phytochemical constituents of salt marshes collected in Palk Bay and Gulf of Mannar waters.

Tests	*Heliotropium curassavicum*		*Sesuvium portulacastrum*		*Suaeda maritima*		*Launaea sarmentosa*		
	Acetone	Ethyl Acetate	Acetone	Ethyl Acetate	Acetone	Ethyl Acetate	Acetone	Ethyl Acetate	Hexane
Alkaloids	++	++	++	++	++	++	++	++	++
Flavonoids	-	++	-	++	++	++	-	++	-
Glycosides	-	++	++	-	++	-	++	++	-
Phenolic groups	-	++	-	-	-	++	-	++	++
Saponins	-	-	-	-	-	-	-	-	++
Steroids	++	++	-	++	++	++	++	++	++
Tannins	++	++	++	++	++	++	++	++	++
Terpenoids	++	++	++	++	++	++	++	++	++
Coumarins	++	-	++	++	++	++	++	++	++
Quinones	++	-	++	-	++	-	++	++	-

“++” indicates the presence, and “-” indicates the absence of phytochemical constituents.

**Table 2:** Zone of inhibition (mm) of salt marsh plants collected during the monsoon season against the candidal strains (CA—*Candida albicans*; CK—*Candida kefyr*; CKr—*Candida krusei;* CT—*Candida tropicalis*; CP—*Candida parapsilosis*).

Sample ID		SM1	SM2	SM3	SM4	SM5	SM6	SM7	SM8	SM9	SM10	Control
**Acetone**	**CA**	10 ± 0.3	10 ± 0.5	10 ± 0.5	9 ± 0.4	10 ± 0.36	10 ± 0.23	-	9 ± 0.21	9 ± 0.55	-	21 ± 0.24
	**CK**	11 ± 0.8	11 ± 0.7	12 ± 0.9	10 ± 0.5	9 ± 0.41	-	9 ± 0.16	10 ± 0.9	9 ± 0.17	10 ± 0.35	18 ± 0.4
	**CKr**	10 ± 0.5	9 ± 0.3	10 ± 0.3	9 ± 0.8	10 ± 0.6	-	-	9 ± 0.6	9 ± 0.8	-	18 ± 0.6
	**CT**	10 ± 0.7	9 ± 0.5	10 ± 0.4	10 ± 0.6	11 ± 0.62	-	-	10 ± 0.9	10 ± 0.9	-	20 ± 0.6
	**CP**	10 ± 0.4	10 ± 0.6	11 ± 0.7	9 ± 0.15	10 ± 0.39	-	-	10 ± 0.57	9 ± 0.7	-	18 ± 0.2
**Ethyl acetate**	**CA**	11 ± 0.76	11 ± 0.36	18 ± 0.45	11 ± 0.78	13 ± 0.59	9 ± 0.37	10 ± 0.32	13 ± 0.87	9 ± 0.6	8 ± 0.3	16 ± 0.6
	**CK**	11 ± 0.6	12 ± 0.74	19 ± 0.59	12 ± 0.36	16 ± 0.7	9 ± 0.4	10 ± 0.71	13 ± 0.35	9 ± 0.7	9 ± 0.4	18 ± 0.3
	**CKr**	10 ± 0.36	10 ± 0.22	20 ± 0.86	12 ± 0.49	13 ± 0.77	-	9 ± 0.33	13 ± 0.25	-	-	21 ± 0.9
	**CT**	10 ± 0.21	11 ± 0.6	19 ± 0.9	12 ± 0.67	13 ± 0.41	10 ± 0.6	10 ± 0.9	15 ± 0.61	9 ± 0.9	9 ± 0.7	19 ± 0.3
	**CP**	12 ± 0.21	13 ± 0.46	18 ± 0.76	12 ± 0.58	15 ± 0.36	-	9 ± 0.36	13 ± 0.28	10 ± 0.57	10 ± 0.33	20 ± 0.41
**Methanol**	**CA**	-	-	9 ± 0.6	-	-	10 ± 0.9	-	-	-	10 ± 0.51	21 ± 0.76
	**CK**	-	9 ± 0.23	-	10 ± 0.12	-	10 ± 0.5	-	10 ± 0.91	-	10 ± 0.84	18 ± 0.6
	**CKr**	10 ± 0.8	-	-	9 ± 0.35	10 ± 0.26	-	-	8 ± 0.2	-	-	20 ± 0.16
	**CT**	9 ± 0.7	9 ± 0.41	9 ± 0.36	9 ± 0.9	-	-	-	-	-	--	18 ± 0.12
	**CP**	-	9 ± 0.22	9 ± 0.5	9 ± 0.17	-	-	-	-	-	10 ± 0.24	21 ± 0.36
**Hexane**	**CA**	-	-	-	-	-	-	-	30 ± 0.23	-	-	21 ± 0.16
	**CK**	-	10 ± 0.33	-	-	8 ± 0.75	-	30 ± 0.95	38 ± 0.71	-	26 ± 0.65	22 ± 0.26
	**CKr**	-	-	-	-	-	9 ± 0.36	-	29 ± 0.46	-	-	18 ± 0.71
	**CT**	-	9 ± 0.14	9 ± 0.5	-	-	10 ± 0.9	-	-	31 ± 0.67	-	20 ± 0.25
	**CP**	-	9 ± 0.7	9 ± 0.62	-	-	-	-	-	27 ± 0.94	-	23 ± 0.8

SM1—*Heliotropium curassavicum*, SM2—*Sesuvium portulacastrum*, SM3—*Suaeda maritima*, SM4—*Ipomoea pes-caprae*, SM5—*Atriplex halimu*, SM6—*Salicornia brachiata*, SM7—*Spinifex littoreus*, SM8—*Launaea sarmentosa*, SM9—*Fimbristylis spathacea*, SM10—*Bulbostylis barbata*. Values with ± denote mean and SD values.

**Table 3:** Zone of inhibition (mm) of salt marsh plants collected during the summer season against the Candidal strains (CA—*Candida albicans*;CK—*Candida kefyr*; CKr—*Candida krusei;* CT—*Candida tropicalis*; CP—*Candida parapsilosis*).

Sample ID		SM1	SM2	SM3	SM4	SM5	SM6	SM7	SM8	SM9	SM10	Control
**Acetone**	**CA**	7 ± 0.6	8 ± 0.8	8 ± 0.4	9 ± 0.9	8 ± 0.45	8 ± 0.61	-	8 ± 0.15	6 ± 0.63	-	18 ± 0.21
	**CK**	9 ± 0.31	9 ± 0.81	9 ± 0.22	6 ± 0.17	7 ± 0.36	-	6 ± 0.32	6 ± 0.21	7 ± 0.16	8 ± 0.37	22 ± 0.4
	**CKr**	8 ± 0.32	8 ± 0.45	8 ± 0.23	10 ± 0.21	9 ± 0.55	-	-	9 ± 0.19	6 ± 0.43	-	21 ± 0.14
	**CT**	10 ± 0.87	8 ± 0.36	8 ± 0.23	9 ± 0.3	9 ± 0.19	8 ± 0.32	-	7 ± 0.6	7 ± 0.4	-	18 ± 0.4
	**CP**	9 ± 0.22	7 ± 0.96	6 ± 0.4	10 ± 0.31	8 ± 0.9	-	-	8 ± 0.7	8 ± 0.13	-	20 ± 0.24
**Ethyl acetate**	**CA**	9 ± 0.18	9 ± 0.3	10 ± 0.8	9 ± 0.63	10 ± 0.16	6 ± 0.25	-	9 ± 0.28	7 ± 0.8	8 ± 0.9	19 ± 0.14
	**CK**	9 ± 0.31	10 ± 0.65	10 ± 0.87	10 ± 0.41	10 ± 0.30	7 ± 0.4	-	10 ± 0.6	8 ± 0.17	9 ± 0.22	20 ± 0.23
	**CKr**	8 ± 0.8	-	-	8 ± 0.37	-	-	-	-	-	-	18 ± 0.41
	**CT**	9 ± 0.21	7 ± 0.12	10 ± 0.38	10 ± 0.44	10 ± 0.9	-	-	10 ± 0.87	9 ± 0.31	-	22 ± 0.44
	**CP**	8 ± 0.33	7 ± 0.11	8 ± 0.41	10 ± 0.36	9 ± 0.25	8 ± 0.9	8 ± 0.17	11 ± 0.9	-	-	21 ± 0.24
**Methanol**	**CA**	-	-	8 ± 0.8	-	-	8 ± 0.7	-	-	-	8 ± 0.2	16 ± 0.4
	**CK**	-	8 ± 0.75	-	9 ± 0.25	-	-	-	8 ± 0.37	-	9 ± 0.9	18 ± 0.14
	**CKr**	-	-	-	8 ± 0.17	9 ± 0	-	-	7 ± 0.14	-	-	15 ± 0.34
	**CT**	-	-	-	8 ± 0.70	-	-	-	-	-	-	19 ± 0.4
	**CP**	-	7 ± 0.64	-	-	-	-	-	-	-	9 ± 0.33	20 ± 0.23
**Hexane**	**CA**	-	-	-	-	-	-	-	24 ± 0.58	-	-	22 ± 0.42
	**CK**	-	-	-	-	8 ± 0.21	-	22 ± 0.87	26 ± 0.78	-	20 ± 0.45	23 ± 0.17
	**CKr**	-	-	-	-	-	-	-	22 ± 0.98	-	-	18 ± 0.29
	**CT**	-	-	8 ± 0.8	-	-	-	-	20 ± 0.47	-	-	19 ± 0.4
	**CP**	-	8 ± 0.70	-	-	-	-	-	-	18 ± 0.37	-	20 ± 0.22

SM1—*Heliotropium curassavicum*, SM2—*Sesuvium portulacastrum*, SM3—*Suaeda maritima*, SM4—*Ipomoea pes-caprae*, SM5—*Atriplex halimu*, SM6—*Salicornia brachiata*, SM7—*Spinifex littoreus*, SM8—*Launaea sarmentosa*, SM9—*Fimbristylis spathacea*, SM10—*Bulbostylis barbata*. Values with ±denote mean and SD values.

**Table 4:** FTIR results obtained from the hexane extract of *Launaea sarmentosa*.

S. No	Absorption (cm^−1^)	Vibration Mode	Compound Class
1	2916.81	N–H stretching	amine salt
2	2849.31	C–H stretching	Alkane
3	2361.41	O=C=O stretching	carbon dioxide
4	1735.62	C=O stretching	Aldehyde
5	1461.78	C–H bending	Alkane
6	1378.85	O–H bending	Phenol
7	1260.25	C–O stretching	aromatic ester
8	1094.4	C–O stretching	secondary alcohol
9	1027.87	C–N stretching	Amine
10	879.381	C=C bending	Alkene
11	802.242	C–H bending	1,4-disubstituted, 1,2,3,4-tetrasubstituted
12	719.318	C=C bending	Alkene

**Table 5:** Compounds present in the hexane extract of *Launaea sarmentosa*, analyzed using GC-MS.

Peaks	RT (min)	Area %	Molecular Weight	Molecular Formula	Compound Names
1	4.442	0.47	163.06	C_6_H_11_Br	Cyclopropane, 2-bromo-1,1,3-trimethyl-
2	6.542	0.33	254.5	C_18_H_38_	Pentadecane, 2,6,10-trimethyl-
3	7.509	0.27	216.38	C_14_H_29_F	Tetradecyl fluoride
4	11.819	0.59	202.33	C_12_H_26_O_2_	1-Methoxy-3-(2-hydroxyethyl)nonane
5	14.463	0.3	138.3	H_6_O_3_Si_3_	Cyclotrisiloxane
6	15.385	0.27	278.5	C_17_H_30_Osi	Trimethyl[4-(1,1,3,3,-tetramethylbutyl)phenoxy]silane
7	15.808	1.08	240.5	C_17_H_36_	Heptadecane
8	16.319	0.5	226.44	C_16_H_34_	Hexadecane
9	16.519	0.28	222.4	C_13_H_22_Osi	Silane, trimethyl[5-methyl-2-(1-methylethyl)phenoxy]-
10	16.819	1.18	282.5	C_20_H_42_	Eicosane
11	17.152	0.39	222.46	C_6_H_18_O_3_Si_3_	Cyclotrisiloxane, hexamethyl-
12	17.296	0.55	250.48	C_13_H_22_OSi_2_	2,4,6-Cycloheptatrien-1-one, 3,5-bis-trimethylsilyl
13	17.53	2.54	366.6	C_24_H_46_O_2_	(Z)-14-Tricosenyl formate
14	17.863	19.03	336.6	C_24_H_48_	Cyclotetracosane
15	18.096	1.07	264.46	C_13_H_20_N_2_SSi	1,2-Benzisothiazol-3-amine tbdms
16	18.418	0.54	222.47	C_12_H_22_Si_2_	Silane, 1,4-phenylenebis[trimethyl
17	18.741	5.32	282.5	C_19_H_38_O	Oxirane, heptadecyl
18	19.107	0.56	346.6	C_20_H42O_2_S	Di-n-decylsulfone
19	19.174	1.04	207.27	C_12_H_17_NO_2_	hexahydropyridine, 1-methyl-4-[4,5 -dihydroxyphenyl]-
20	19.485	0.55	264.43	C_15_H_24_O_2_Si	Trimethyl[4-(2-methyl-4-oxo-2-pentyl)phenoxy]silane
21	20.374	6.4	268.5	C_18_H_36_O	Octadecanal
22	20.651	0.85	310.68	C_10_H_30_O_3_Si_4_	Tetrasiloxane, decamethyl-
23	21.007	0.41	242.65	C_14_H_7_ClO_2_	9-Fluorenone-4-carbonyl chloride
24	21.285	0.61	250.38	C_16_H_26_O_2_	Hexanoic acid, 2,7-dimethyloct-7-en-5-yn-4-yl ester
25	21.507	2.97	218.33	C_15_H_22_O	2(1H)Naphthalenone, 3,5,6,7,8,8a-hexahydro-4,8a-dimethyl-6-(1-methylethenyl)-
26	21.862	12.66	426.7	C_30_H_50_O	β-amyrin
27	22.051	3.71	218.25	C_16_H_10_O	Benzo[b]naphtho[2,3-d]furan
28	22.429	24.16	426.7	C_30_H_50_O	Lupeol
29	22.618	1	310.68	C_10_H_30_O_3_Si_4_	Methyltris(trimethylsiloxy)silane
30	22.873	10.35	468.8	C_32_H_52_O_2_	12-Oleanen-3-yl acetate

**Table 6:** The location of salt marshes collected from in the study area.

Location	Sites	Latitude	Longitude
Rameswaram	Site 1	9°16′54.5″ N	79°11′04.5″ E
	Site 2	9°16′55.2″ N	79°11′19.3″ E
	Site 3	9°16′52.2″ N	79°11′21.4″ E
Dhanushkodi	Site 4	9°12′07.2″ N	79°22′38.9″ E
Rameswaram	Site 5	9°17′02.1″ N	79°10′41.5″ E
Dhanushkodi	Site 6	9°10′04.6″ N	79°25′46.0″ E
	Site 7	9°09′58.7″ N	79°25′53.2″ E
	Site 8	9°09′56.7″ N	79°25′54.3″ E

**Table 7:** Distribution of salt marsh plants in the study area.

Sample ID	Name of the Species	Site 1	Site 2	Site 3	Site 4	Site 5	Site 6	Site 7	Site 8
SM1	*Heliotropium curassavicum*	+							
SM2	*Sesuvium portulacastrum*	+							
SM3	*Suaeda maritima*	+							
SM4	*Ipomoea pes-caprae*		+						
SM5	*Atriplex halimus*			+					
SM6	*Salicornia brachiata*				+				
SM7	*Spinifex littoreus*					+			
SM8	*Launaea sarmentosa*						+		
SM9	*Fimbristylis spathacea*							+	
SM10	*Bulbostylis barbata*								+

“+” indicates the availability of salt marsh plants in sampling sites.

## Data Availability

The data that support the findings of this study are available from the corresponding author upon reasonable request.

## Supplementary Materials

The following supporting information can be downloaded at https://www.mdpi.com/article/10.3390/antibiotics13080748/s1, Supplementary Figure S1 Shows the zone of inhibition (mm) based on the methanol solvent extract from 10 different salt marshes species. Supplementary Figure S2 shows Zone of inhibition (mm) based on the hexane solvent extract from salt marshes. Supplementary Figure S3 shows the Zone of inhibition (mm) based on the acetone solvent extract from salt marshes. Supplementary Figure S4 shows the Zone of inhibition (mm) based on the ethyl acetate solvent extract from salt marshes. Supplementary Figure S5A,B shows the phytochemical analysis acetone and ethyl acetate extract of SM1-*Heliotropium curassavicum*. Supplementary Figure S6A,B shows the phytochemical analysis acetone and ethyl acetate extract of SM2-*Sesuvium portulacastrum*. Supplementary Figure S7A,B show the phytochemical analysis acetone and ethyl acetate extract of SM3-*Suaeda maritima*. Supplementary Figure S8A–C show the phytochemical analysis acetone, ethyl acetate and hexane extract of SM8-*Launaea sarmentosa*.
