# Supplementary material for: Anticandidal Properties of Launaea sarmentosa among the Salt Marsh Plants Collected from Palk Bay and the Gulf of Mannar Coast, Southeastern India
Source: Antibiotics (Basel). 2024 Aug 9;13(8):748. doi: 10.3390/antibiotics13080748 (PMC11350644; doi:10.3390/antibiotics13080748)

SUPPLEMENTARY DATA

Anticandidal Properties of *Launaea sarmentosa* among the Salt Marsh Plants Collected from Palk Bay and the Gulf of Mannar Coast, Southeastern India

Smriti Das [1,†], Karuppannagounder Rajan Priyanka [1], Kolandhasamy Prabhu [1], Ramachandran Vinayagam [2,†], Rajendran Rajaram [1,*] and Sang Gu Kang [2,*]

[1] Department of Marine Science, School of Marine Sciences, Bharathidasan University, Tiruchirappalli 620 024, Tamil Nadu, India; dassmriti874@gmail.com (S.D.); priyanka.kr@bdu.ac.in (K.R.P.); prabhu.k@bdu.ac.in (K.P.)

[2] Department of Biotechnology, Institute of Biotechnology, College of Life and Applied Sciences, Yeungnam University, 280 Daehak-Ro, Gyeongsan 38541, Gyeongsangbuk-do, Republic of Korea; rambio8@gmail.com or rambio@ynu.ac.kr

* Correspondence: rajaramdms@bdu.ac.in (R.R.); kangsg@ynu.ac.kr (S.G.K.)

† These authors contributed equally to this work.

Figure S1. The zone of inhibition (mm) based on the methanol solvent extract from 10 different salt marsh species (CA—*Candida albicans*; CR—*Candida kefyr*; CKr—*Candida krusei*; CT—*Candida tropicalis*; CP—*Candida parapsilosis*). SM5–*Atriplex halimu*; SM10–*Bulbostylis barbata*

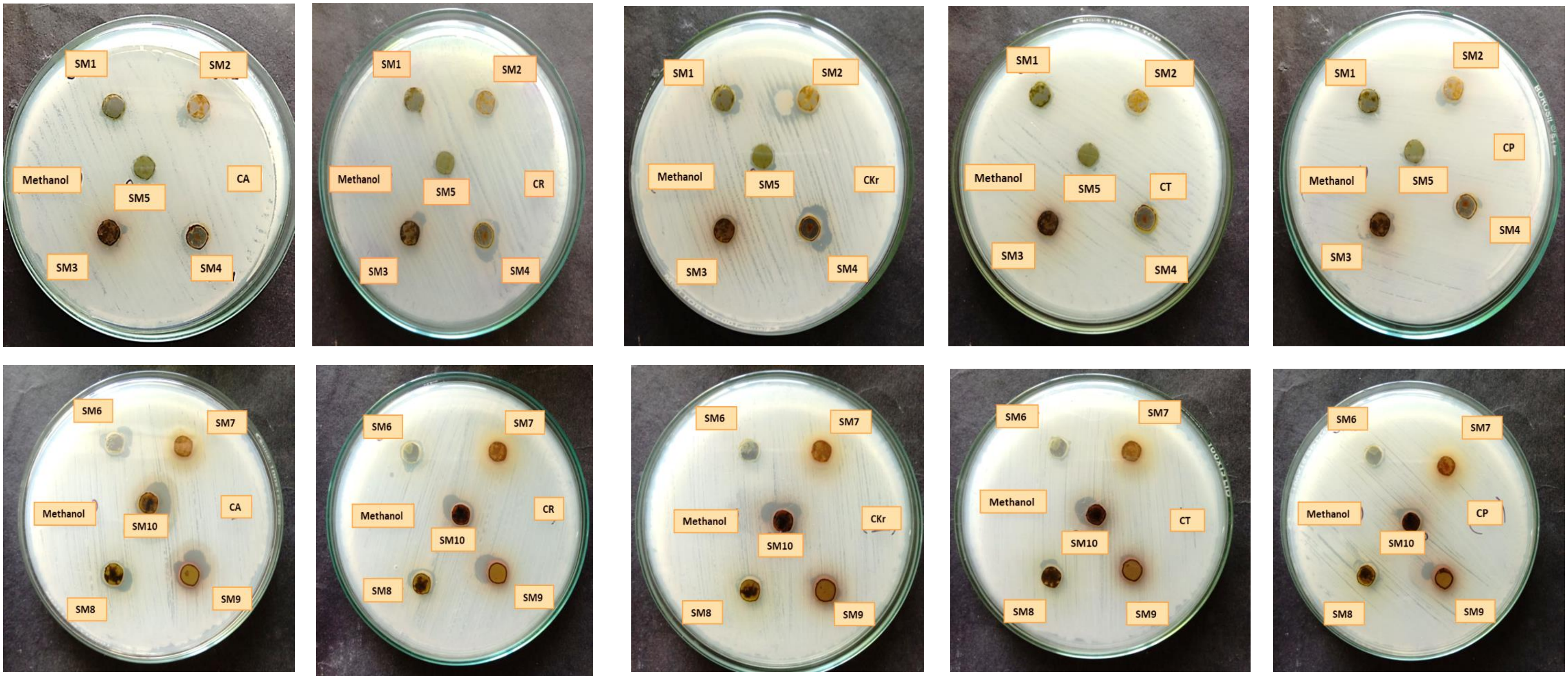

Figure S2. The zone of inhibition (mm) based on the hexane solvent extract from salt marshes (CA—*Candida albicans*; CR—*Candida kefyr*; CKr—*Candida krusei*; CT—*Candida tropicalis*; CP—*Candida parapsilosis*) SM1—*Heliotropium curassavicum*; SM2—*Sesuvium portulacastrum*; SM3—*Suaeda maritima*; SM4—*Ipomoea pes-caprae*; SM5—*Atriplex halimu*; SM6—*Salicornia brachiata*; SM7—*Spinifex littoreus*.

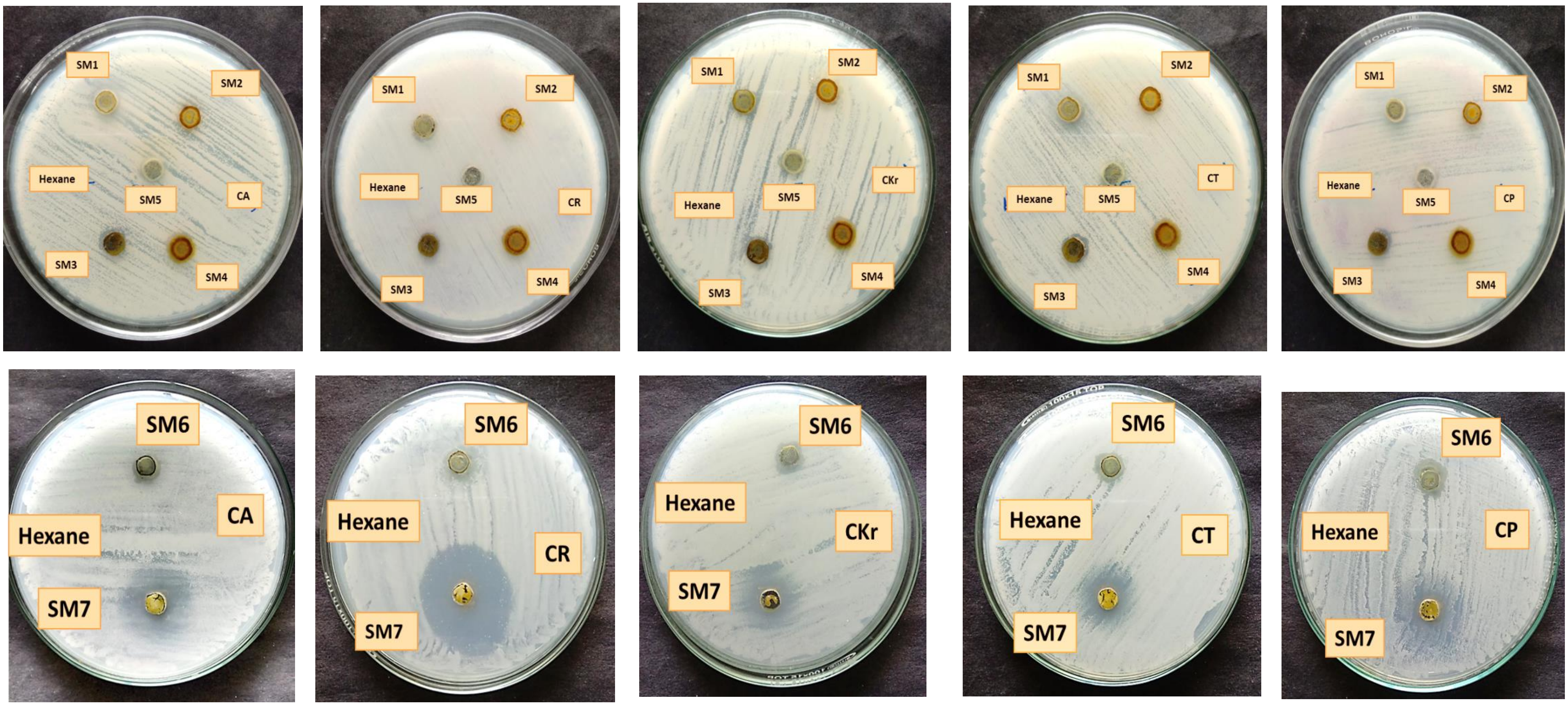

Figure S3. The zone of inhibition (mm) based on the acetone solvent extract from salt marshes (CA—*Candida albicans*; CR—*Candida kefyr*; CKr—*Candida krusei*; CT—*Candida tropicalis*; CP—*Candida parapsilosis*). SM1—*Heliotropium curassavicum*; SM2—*Sesuvium portulacastrum*; SM3—*Suaeda maritima*; SM4—*Ipomoea pes-caprae*; SM5—*Atriplex halimu*; SM6—*Salicornia brachiata.*

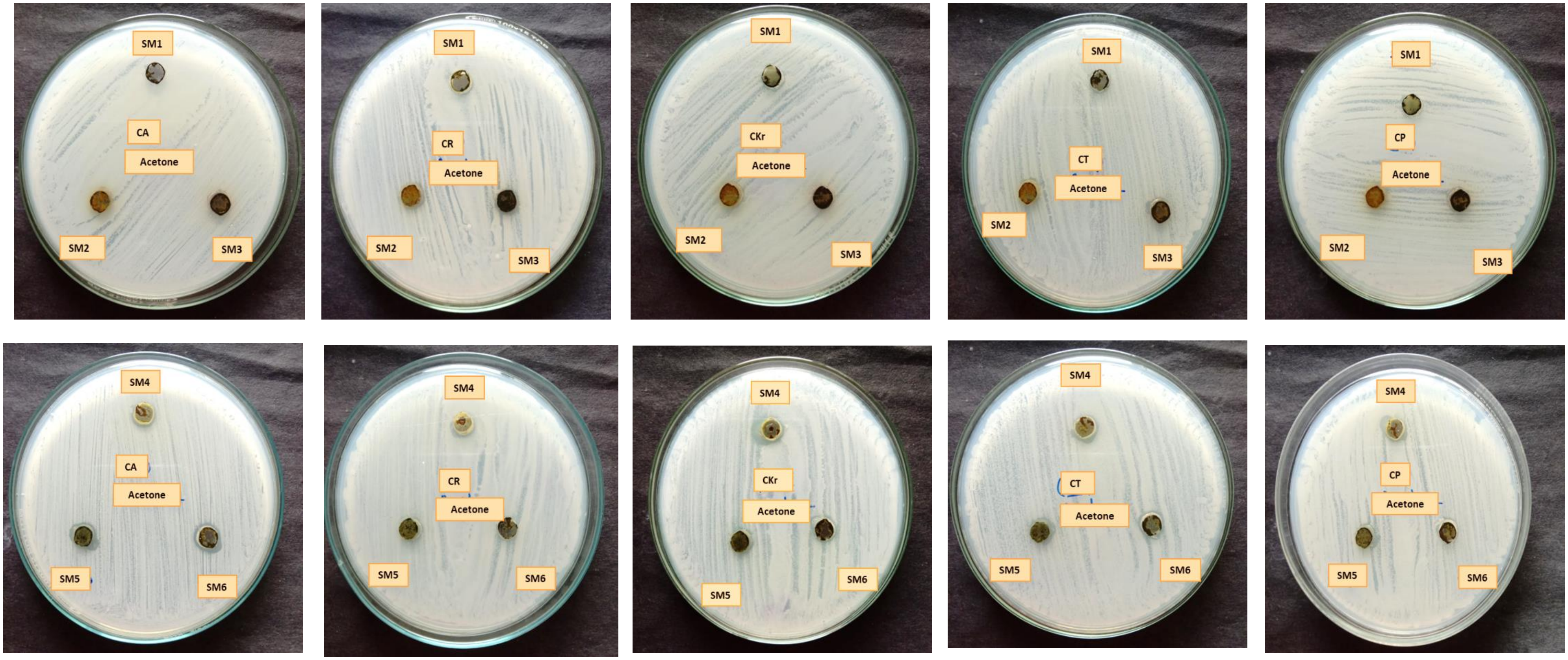

Figure S4. The zone of inhibition (mm) based on the ethyl acetate solvent extract from salt marshes (CA—*Candida albicans*; CR—*Candida kefyr*; CKr—*Candida krusei*; CT—*Candida tropicalis*; CP—*Candida parapsilosis*) SM1—*Heliotropium curassavicum*; SM2—*Sesuvium portulacastrum*; SM3—*Suaeda maritima*; SM4—*Ipomoea pes-caprae*; SM5—*Atriplex halimu*; SM6—*Salicornia brachiata*; *SM7—Spinifex littoreus*; *SM8—Launaea sarmentosa*; *SM9—Fimbristylis spathacea*; *SM10—Bulbostylis barbata.*

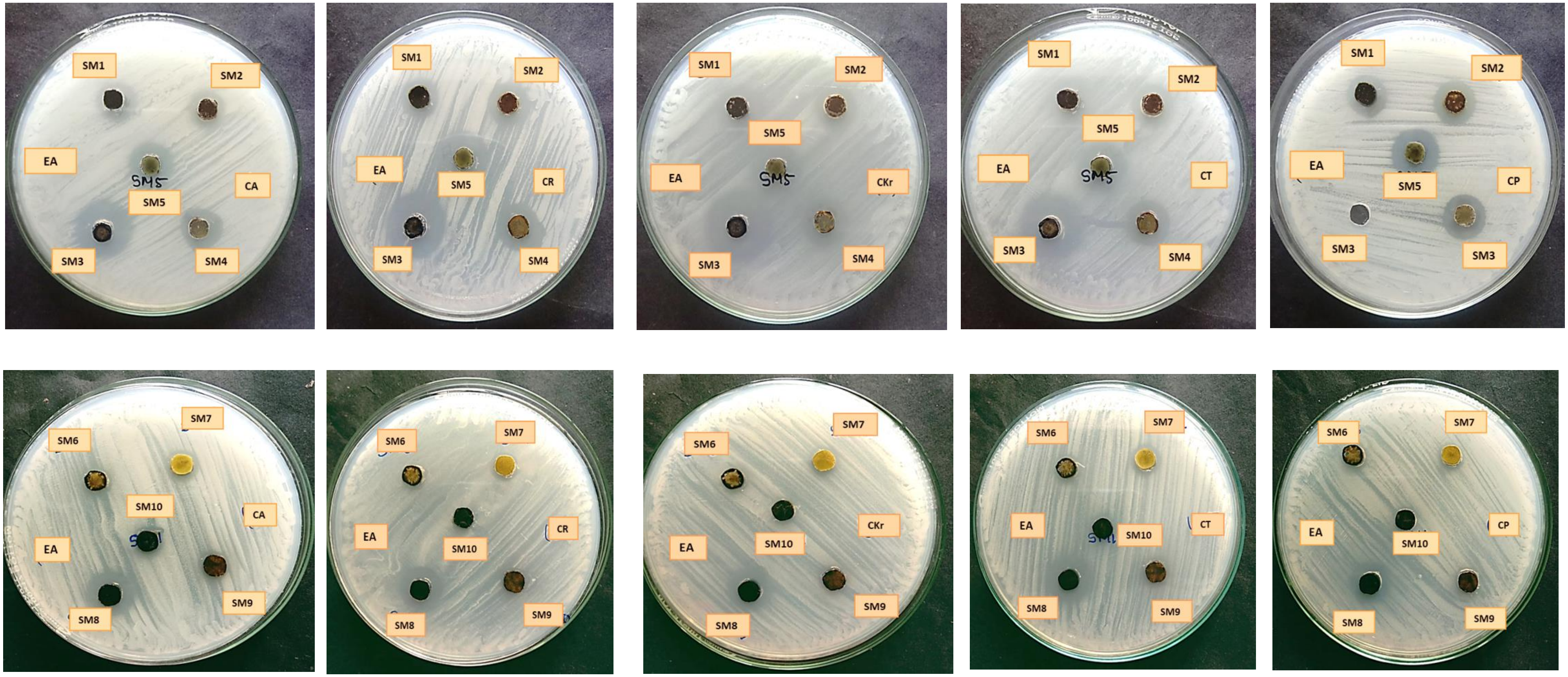

Figure S5. A and B show the phytochemical analysis of the acetone and ethyl acetate extracts of SM1—*Heliotropium curassavicum*.

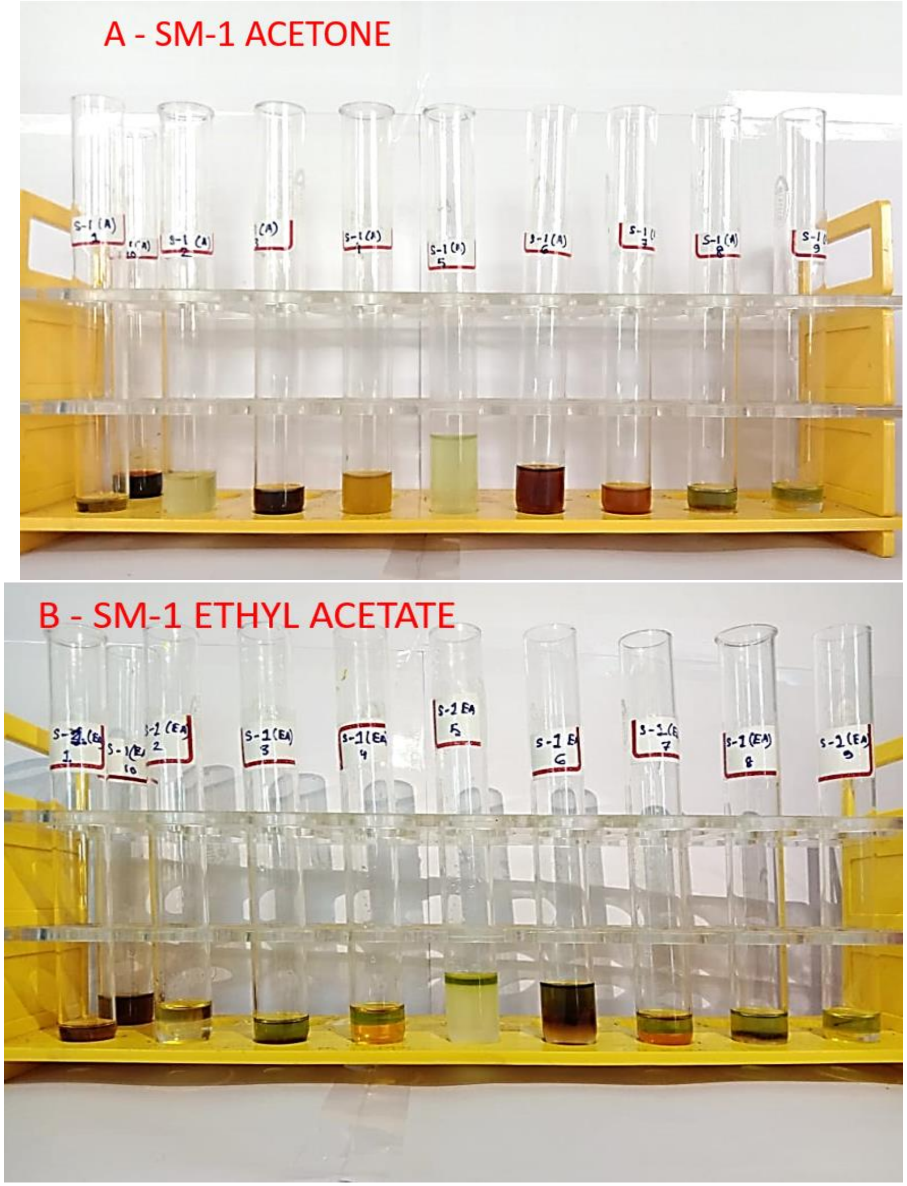

Figure S6. A and B show the phytochemical analysis of the acetone and ethyl acetate extracts of SM2—*Sesuvium portulacastrum*.

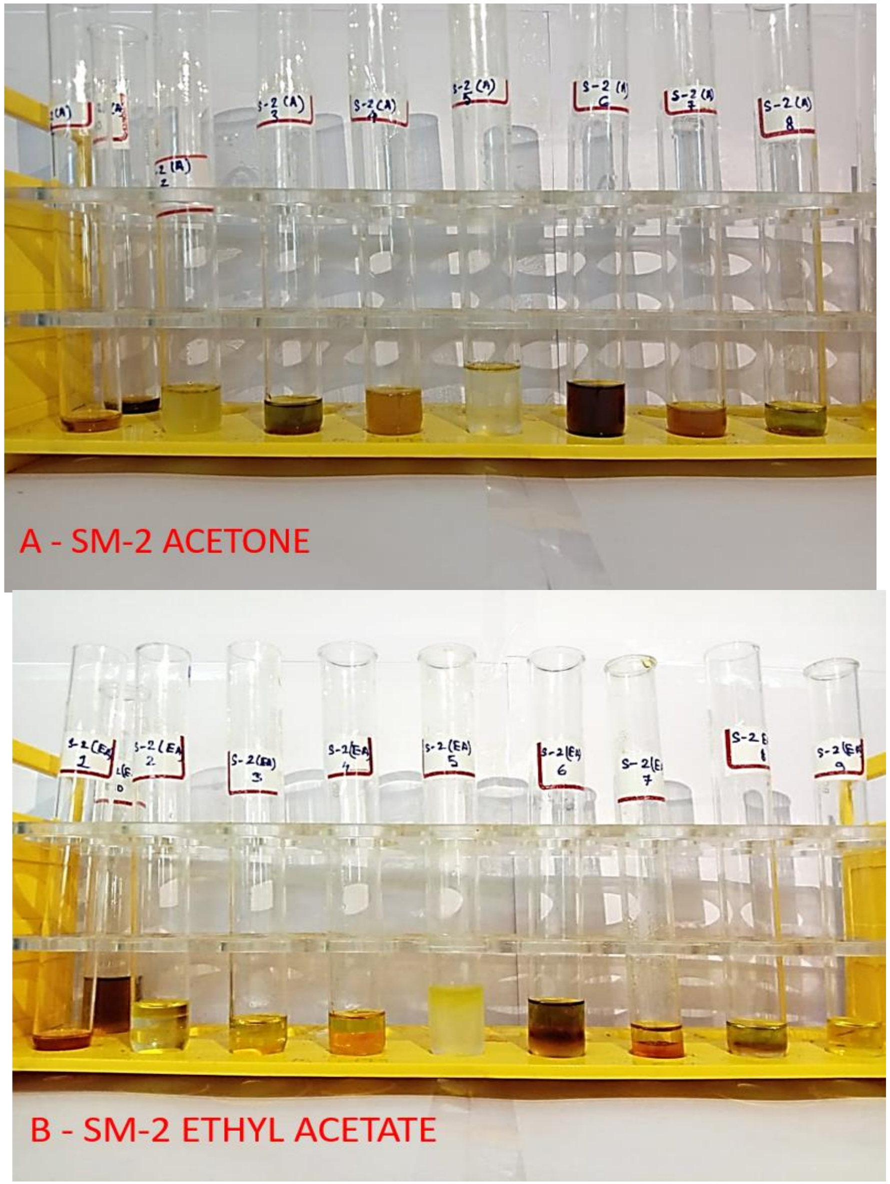

Figure S7. A and B show the phytochemical analysis of the acetone and ethyl acetate extracts of SM3—*Suaeda maritima*.

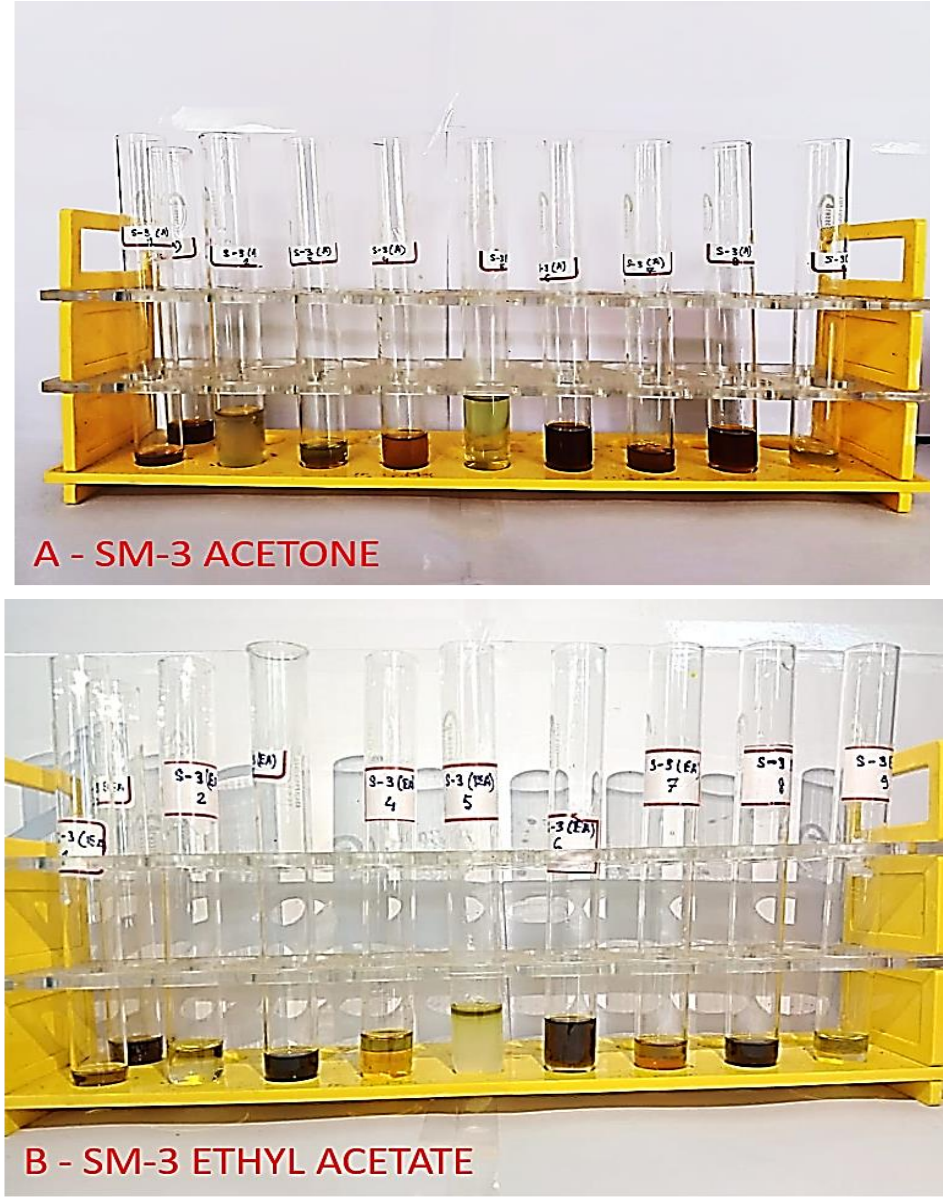

Figure S8. A, B, and C show the phytochemical analysis of the acetone, ethyl acetate, and hexane extracts of *SM8—Launaea sarmentosa*

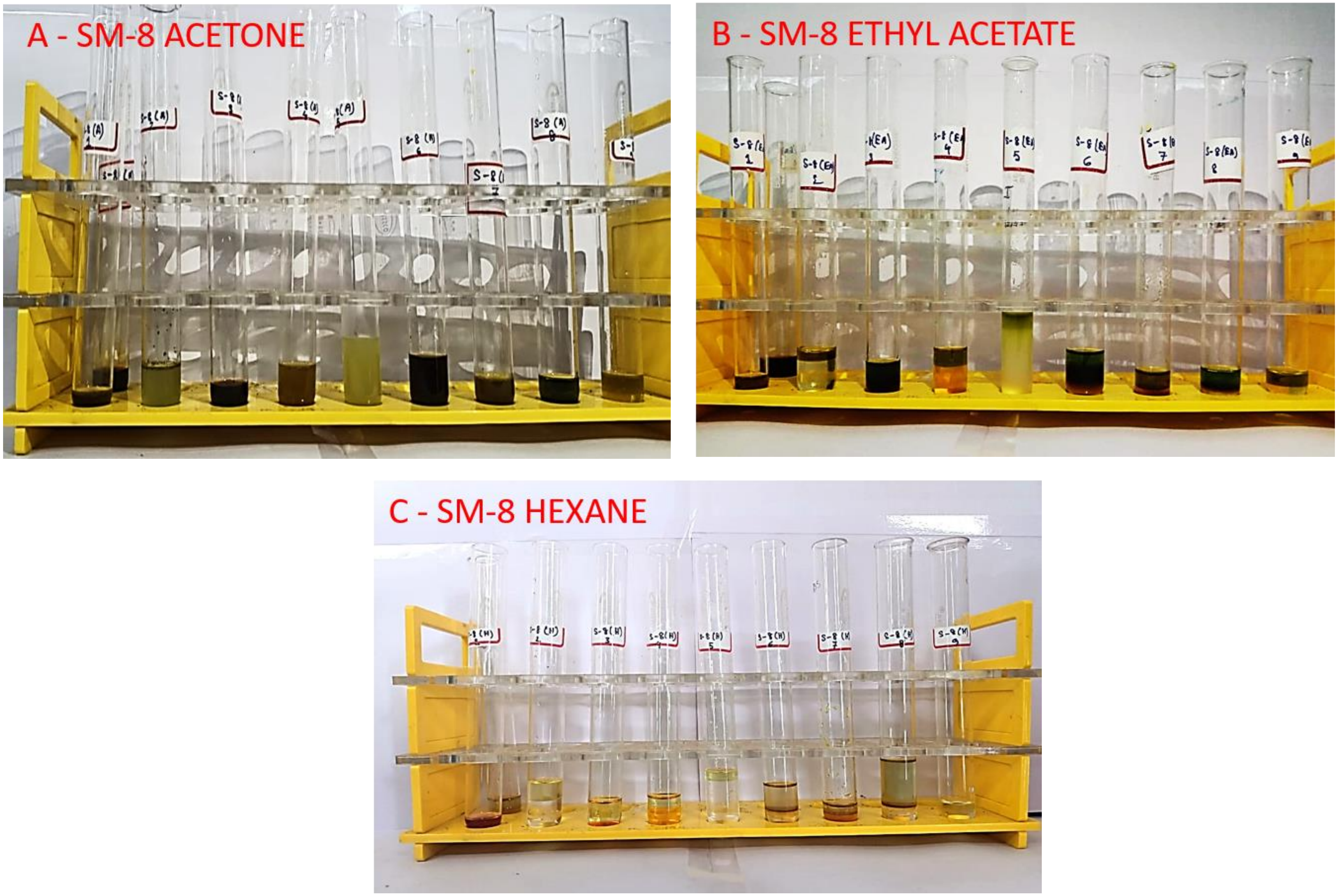

Supplement: Supplementary file 1 [file antibiotics-13-00748-s001.zip › antibiotics-3104294-supplementary.pdf]
